# Security by design in artificial intelligence-enabled energy management systems: a sociotechnical framework

**DOI:** 10.3389/frai.2026.1867922

**Published:** 2026-06-26

**Authors:** Theodore Kindong, Gianluigi Viscusi, Björn Johansson

**Affiliations:** Linköping University, Linköping, Sweden

**Keywords:** artificial intelligence, design science research, smart grid, energy management systems, security

## Abstract

The global energy sector is undergoing a profound digital transformation driven by growing demands for sustainability, efficiency, and resilience. The shift from traditional power grids to smart, decentralized energy systems has accelerated the adoption of artificial intelligence (AI)-enabled Energy Management Systems (EMS). While these technologies offer significant benefits, they also introduce new data security and governance challenges that must be addressed to ensure trustworthy and resilient energy infrastructure. This study adopts a Design Science Research (DSR) approach to investigate security issues associated with the development and implementation of AI-enabled EMS. Through an analysis of academic and practice-oriented literature, a conceptual framework was developed that takes a lifecycle perspective, tracing data flows through interconnected phases of *data generation*, *sensing*, *model development*, and *deployment.* Based on the identified challenges, four design principles were formulated to address specific security vulnerabilities. The primary outcome is a conceptual artifact that categorizes data security challenges in AI-enabled EMS across different lifecycle phases. The analysis revealed that these challenges require coordinated action by multiple stakeholders across different system layers, supported by dedicated security tools and governance mechanisms. The study proposes the *Prevent*–*Audit*–*Learn*–*Harden* (PALH) framework as a practical approach for guiding the secure design, deployment, and operation of AI-enabled EMS. By linking identified security challenges to corresponding design principles and lifecycle stages, the framework helps strengthen data security and governance in smart energy systems. The findings highlight the importance of a holistic, lifecycle-oriented approach to security in supporting the ongoing transition toward decentralized smart grids.

## 1 Introduction

The global energy sector is undergoing a profound digital transformation driven by increasing demands for sustainability, efficiency, and resilience. This transformation has led to a decentralized grid defined as a smart grid. Smart grids represent the evolution of traditional power grids through the integration of digital communication technologies, distributed energy resources, and advanced analytics (El-Hawary, 2016; Zohuri, 2023). More so, this continuous transformation has seen the integration of advanced technologies, with technologies such as artificial intelligence (AI) being increasingly embedded in Energy Management Systems (EMS) to enable predictive maintenance, demand response, autonomous control, and optimization of energy generation and distribution (Mohammadi et al., 2022; Yan et al., 2019). This transforms the traditional power grid into an AI-enabled smart grid, positioning it as a core component of intelligent cities and next-generation cyber-physical infrastructures.

However, when integrating AI it transforms smart grids into highly complex cyber-physical and socio-technical systems, introducing new data security and governance challenges. At the same time has AI-enabled cyber-physical systems demonstrated significant potential for enhancing resilience in smart environments, for instance as, decentralized autonomous vehicles acting as first responders in smart cities (Majumdar et al., 2025). Similarly, AI-enabled EMS promises improved performance and efficiency in energy infrastructures (Boopathy et al., 2024; Natarajan et al., 2025). Yet, the reliance on large-scale data collection, distributed Internet of Things (IoT) devices, cloud and edge computing, and autonomous decision-making significantly expands the attack surface and introduces vulnerabilities that extend beyond traditional cybersecurity threats.

Historically, energy infrastructure security research has focused on physical, geopolitical, and reliability risks (Farrell et al., 2004), ignoring contemporary smart grids, which often introduce cyber, data-driven, and AI-specific vulnerabilities, including data poisoning, adversarial attacks, model extraction, privacy leakage, and lifecycle management risks (Bekara, 2014; Khan et al., 2023). In addition, smart grids are inherently socio-technical systems involving multiple stakeholders, including utilities, regulators, technology providers, and consumers, making organizational governance and regulatory compliance integral to system security (Herath et al., 2020; Kosenkov et al., 2025).

Moreover, despite extensive research on smart grid cybersecurity and AI applications, existing studies predominantly focus on technical mechanisms and performance improvements. Hence, there remains a lack of prescriptive design knowledge that integrates AI security mechanisms with organizational governance, regulatory requirements, and socio-technical system design. From an Information Systems perspective, such prescriptive knowledge is essential for guiding the design and implementation of trustworthy AI-enabled critical infrastructures. To address this gap, we conduct a Design Science Research (DSR) project (Gregor and Jones, 2007; Hevner et al., 2004; Peffers et al., 2007) to develop actionable design knowledge (Goldkuhl, 2013) for securing AI-enabled Energy Management Systems. Particularly, we explore how new actors, such as Energy Management Systems (EMS) providers, and development and implementation of EMS influence security issues. Accordingly, the project is guided by the following research questions:

- What security issues are of concern when developing and implementing AI-enabled energy management systems?
- How can they be addressed through prescriptive design knowledge?

In response to these research questions, this paper presents, in the theoretical background, the results of the first stages of the ongoing DSR, focusing on problem identification, motivation, and the definition of the objectives for the suggested artifact for the solution (Peffers et al., 2007). In those stages, we identify the kernel theories (Möller et al., 2022) to understand the problem, define meta-requirements, and identify success criteria for the artifact (Gregor and Jones, 2007; Walls et al., 1992). Accordingly, we conduct a scoping literature review (Munn et al., 2018; Veloso and Varajão, 2025) to identify key data security challenges and derive design requirements and design principles for secure AI-enabled EMS. Then, the paper presents the results of the artifact design (Peffers et al., 2007) as a conceptual socio-technical framework that integrates technical security mechanisms with governance and human oversight structures. The study contributes to Information Systems and AI literature by extending design theory to AI-enabled critical infrastructures and by guiding researchers, practitioners, and policymakers involved in the digital transformation of energy systems.

## 2 Theoretical background

To identify and synthesize existing knowledge on data security challenges in AI-enabled smart grid systems, this DSR project employed a scoping review approach following the framework proposed by Arksey and O’Malley (2005). This approach was chosen because it is well-suited to emerging or interdisciplinary research areas where knowledge is fragmented and understanding is still developing. Therefore, the purpose of the scoping review was to map the key concepts, research themes, and knowledge gaps within our topic of interest: security in AI-enabled smart grids. The review process followed five main stages as outlined by Arksey and O’Malley (2005): (i) Identifying the research question, (ii) Identifying relevant studies, (iii) Study selection, (iv) Charting the data, and (v) Collating and synthesizing the results. After defining the research questions, the authors searched for relevant literature through major academic databases commonly used in information systems and engineering research. This include an advanced searches in Google Scholar using combined keywords such as “smart grids,” “artificial intelligence,” “AI-enabled energy management systems,” “cybersecurity,” and “data security,” across specific fields, including “all of the words” to capture core terms (artificial intelligence applications in smart grids), “exact phrase” to ensure precise matches, and “at least one word” to broaden results with related terms. The “without the words” option was applied to exclude irrelevant topics, while the “where my words occur” filter (AI-enabled energy management systems) was used to improve the relevance of results. Moreover, advanced searches using combinations of the same keywords as in Google Scholar, with the “AND” and “OR” operators, were conducted to retrieve relevant literature from the AIS eLibrary, IEEE Xplore, and the ACM Digital Library. Also, publication year and “cited by” filters were used to limit the results to a defined timeframe, and duplicate records across databases were identified and removed prior to screening. After identifying relevant literature, the inclusion criteria were defined as peer-reviewed articles focused on AI applications in smart grid systems and their socio-technical and cybersecurity implications, and non-peer-reviewed articles, articles unrelated to energy systems, or those lacking technical or socio-technical relevance were excluded.

### 2.1 Smart grids as socio-technical energy information systems

Smart grids represent a transformation of traditional electricity infrastructures into digitally enabled energy ecosystems that integrate information technologies, distributed energy resources, and intelligent control mechanisms (Dhara et al., 2022). Smart grids can therefore be understood as socio-technical systems, in which technological infrastructures, organizational actors, institutional arrangements, and user practices interact to jointly shape system outcomes (De Wilde et al., 2016; Mah et al., 2012). From this it can be suggested that, system performance depends on alignment between technical subsystems (e.g., digital infrastructure, smart meters, AI algorithms) and social subsystems (e.g., regulators, operators, and consumers) (Bostrom and Heinen, 1977; Dwyer, 2011), especially since socio-technical systems theory emphasizes the principle of joint optimization. From this it can be argued that technologically sophisticated systems may fail when organizational structures, governance mechanisms, and user practices are not adequately aligned (Bostrom and Heinen, 1977).

Moreover, the transformation of traditional electricity infrastructures toward digitally enabled smart grids integrates advanced communication networks, distributed energy resources, and intelligent control technologies, making the smart grid a purely socio-technical system. Fundamentally, the transformation reshapes how electricity systems are designed, managed, and governed (De Wilde et al., 2016). Thus, smart grids move from operating as centralized technical infrastructures to increasingly function as digitally interconnected socio-technical networks involving utilities, regulators, consumers, and technology providers. The transition from centralized to decentralized structure also involves significant institutional and governance challenges, since development of smart grid infrastructures is influenced not only by technological innovation but also by regulatory frameworks, policy incentives, and stakeholder coordination (Mah et al., 2012). Consequently, the implementation of digital energy technologies requires coordinated changes across technological architectures, organizational processes, and policy environments.

From an information systems perspective, adoption of digital technologies in the electricity sector further illustrates the socio-technical nature of smart grid systems. This indicates that the diffusion and adoption of smart metering technologies are strongly influenced by factors such as consumer trust, perceived privacy risks, and regulatory legitimacy (Wunderlich et al., 2012). Thus, digital energy technologies must be designed with consideration for both technical performance and social acceptance. It also indicates that the socio-technical perspective has been widely applied in research on sustainability and environmental information systems to analyze how digital technologies support transitions toward more sustainable infrastructures. Resulting in a smart grid, which constitutes Green ICT initiatives, illustrating how digital systems can enable environmentally sustainable socio-technical transitions through improved resource management and energy efficiency (Butler et al., 2015).

It can also be claimed that the growing digitalization of energy infrastructures has led to an increase in adoption of artificial intelligence (AI) technologies within energy management systems. Different AI techniques are being applied to optimize electricity demand forecasting, energy storage management, renewable energy integration, and microgrid coordination (Mohammadi et al., 2022; Marques and Oliveira, 2024). These technologies enable energy systems to operate more efficiently and support the transition toward decentralized and sustainable electricity networks. Fostering decentralized decision-making mechanisms capable of enhancing grid stability while accommodating diverse user behaviors (Pournaras et al., 2019). Such approaches illustrate how technological optimization strategies must be integrated with behavioral dynamics of distributed energy participants. However, it can also be claimed that integration of digital technologies and AI into energy infrastructures introduces new operational and governance challenges. The reason is that AI-driven smart energy services require reliable data infrastructures, advanced analytics capabilities, and coordinated decision-making processes across multiple stakeholders (Gerlach et al., 2023). More so, the integration of AI into business processes within the electricity sector is transforming operational workflows and decision-making structures, further increasing the complexity of energy management system (Tchatchouang Wanko, 2025). From this it can be stated that it is important to provide a robust theoretical foundation for examining data security issues in AI-enabled energy management systems and for developing prescriptive design knowledge that supports secure and trustworthy smart grid operations. As energy infrastructures become increasingly digitalized and interconnected, ensuring the security and resilience of these socio-technical systems becomes a critical challenge. The following section, therefore, examines the role of cybersecurity-by-design approaches in protecting smart grid infrastructures.

### 2.2 Cybersecurity-by-design in smart grids

The digital transformation of energy infrastructures has significantly expanded the cyber-attack surface of electricity networks. This is because smart grids rely on interconnected communication networks, Internet-of-Things (IoT) devices, advanced metering infrastructures, and cloud-based data platforms to coordinate energy production and consumption. While these technologies enable greater efficiency and operational flexibility, they also introduce new cybersecurity risks, such as communication attacks, data manipulation, identity spoofing, and vulnerabilities in available assets that can threaten the stability and reliability of critical energy infrastructure (Bekara, 2014; Dalipi and Yayilgan, 2016). These security issues continue to emerge, with recent literature highlighting advanced threats, including adversarial AI/ML attacks, data poisoning, and vulnerabilities that can compromise decision-making processes (Kwon et al., 2025; Sánchez et al., 2024; Zhang et al., 2024). These findings indicate that security issues in smart grids are increasingly complex with the adoption of AI applications, spanning both technical weaknesses in learning models and broader socio-technical dependencies within smart grid systems. Thus, deploying secure AI applications requires adopting a cybersecurity-by-design approach. Cybersecurity research increasingly emphasizes the importance of cybersecurity-by-design, which integrates security mechanisms directly into system architectures during the development phase rather than addressing vulnerabilities only after system deployment (Bathalapalli et al., 2025; De Kinderen et al., 2022; Fridgen et al., 2022; Gerlach et al., 2022). This approach is particularly important for critical infrastructures such as smart grids, where system disruptions can have significant economic and societal consequences.

Smart grid architectures consist of multiple interconnected components, including advanced metering infrastructures (AMI), distributed sensors, communication networks, and energy management platforms (Al Dakhl et al., 2023; Dhara et al., 2022). Each of these components introduces potential security vulnerabilities. For example, advanced metering infrastructures play a central role in enabling real-time monitoring of energy consumption but are also vulnerable to cyberattacks that target data integrity and system availability (Bendiab et al., 2020). Similarly, smart meters themselves may contain security weaknesses that expose energy systems to unauthorized access, data manipulation, and service disruption (Khattak et al., 2019). The increasing use of IoT technologies within smart grids further expands the potential attack surface. IoT devices enable distributed monitoring and automated control of energy systems, but often operate with limited computational resources and security capabilities, making them attractive targets for cyber attackers (Dalipi and Yayilgan, 2016; Kimani et al., 2019). As a result, ensuring the security of IoT-based energy infrastructures requires comprehensive security frameworks that address vulnerabilities across device, network, and application layers.

To address these challenges, researchers and governments have proposed structured cybersecurity frameworks and standards that guide the design of secure smart grid architectures. Mapping and aligning cybersecurity standards in smart grids is particularly important because energy infrastructure often integrates heterogeneous technologies from multiple vendors and organizations. Niemann et al. (2024) highlight the growing need for coordinated cybersecurity standards that guide secure smart grid infrastructures across different technical and regulatory contexts. Comprehensive cybersecurity frameworks have therefore been proposed to ensure dynamic protection of cyber-physical systems operating within smart grid environments (Abi Sen, 2022). Thus, Regulatory frameworks such as the EU AI Act (EU, E., 2024) and the NIS2 Directive (European Commission, E. C., 2022) have emerged as regulatory sandboxes, providing overarching guidance for the design and deployment of technology solutions, especially in modern energy systems. These regulations require energy providers and operators of critical infrastructure to implement robust security, risk management, and governance mechanisms to protect sensitive consumer and operational data and to ensure the trustworthy, secure deployment of AI-driven analytics and decision-making systems. In particular, the EU AI Act emphasizes transparency, accountability, and risk mitigation for high-risk AI applications, whereas NIS2 strengthens cybersecurity resilience, incident reporting, and operational security requirements across essential sectors, including energy.

In addition to governance and regulatory frameworks, technological innovations are increasingly being explored to enhance smart grid security. Blockchain technologies, for example, have been proposed as mechanisms for secure energy transactions and ensuring data integrity within distributed energy management systems (Khan et al., 2023; Kim et al., 2019). These technologies can help establish trust among multiple actors participating in decentralized energy markets. While cybersecurity-by-design provides essential architectural and governance principles, the growing adoption of artificial intelligence within energy systems introduces additional security considerations. AI-enabled energy management systems rely heavily on large volumes of data and complex machine learning models, which introduce new potential vulnerabilities related to data manipulation and algorithmic attacks. The following section, therefore, examines the data security challenges associated with AI-enabled energy management systems in smart grid environments.

### 2.3 AI-enabled energy management systems and data security issues

Artificial intelligence is increasingly being integrated into energy management systems to support advanced decision-making capabilities in smart grid environments. AI technologies enable predictive demand forecasting, automated demand response, grid stability management, and optimization of distributed energy resources. These capabilities allow energy systems to respond dynamically to fluctuations in energy demand and supply while improving overall grid efficiency. AI-enabled energy management systems rely on diverse datasets collected from smart meters, environmental sensors, energy markets, and user behavior patterns. These data sources enable advanced machine learning models that support intelligent energy management and operational optimization (Khan et al., 2022). In microgrid environments, AI techniques are increasingly used to coordinate distributed energy resources and optimize power generation and storage strategies (Mohammadi et al., 2022).

Recent research also highlights the potential of AI technologies to significantly enhance smart grid performance. AI-driven optimization algorithms can improve energy efficiency, support renewable energy integration, and enhance grid stability (El Rhatrif et al., 2024; Kiliç et al., 2024). Empirical studies further suggest that AI-enabled energy management systems can improve operational performance and support more sustainable energy systems (Natarajan et al., 2025). However, the growing reliance on data-driven decision-making processes introduces new cybersecurity risks. AI models deployed in smart grids are exposed to multiple, well-documented cyberattack vectors that can compromise both performance and operational reliability. Data poisoning attacks target the training phase by injecting malicious or biased data, leading to systematic flaws in model behavior and degraded prediction accuracy in downstream tasks such as load forecasting or control optimization (Sánchez et al., 2024; Zhang et al., 2024). Similarly, adversarial manipulation occurs during inference, where carefully crafted input perturbations, often imperceptible, can mislead models into producing incorrect outputs, posing risks to real-time grid stability and decision-making (Kwon et al., 2025). Additionally, model extraction attacks exploit query access to reconstruct or approximate the underlying model, potentially exposing sensitive system logic and enabling further targeted attacks (Sánchez et al., 2024). These vulnerabilities are amplified in IoT-enabled and distributed grid environments, where heterogeneous data sources, limited device security, and communication constraints increase the attack surface (Zhang et al., 2024). Reinforcement learning-based control systems introduce new risks due to their continuous interaction with the environment and reliance on reward signals, which can be manipulated to induce unsafe policies (Kwon et al., 2025). Consequently, understanding and mitigating these threats is critical for ensuring the robustness, reliability, and trustworthiness of AI-driven smart grid applications. These vulnerabilities are particularly concerning in critical infrastructures such as smart grids, where compromised decision-making systems can disrupt electricity supply or manipulate energy markets.

Emerging approaches such as federated learning have been proposed to address some of these challenges by enabling distributed machine learning without centralizing sensitive data. Federated learning allows multiple entities to collaboratively train machine learning models while keeping local datasets private. While this approach offers potential benefits for privacy-preserving energy analytics, it also introduces new security challenges related to model integrity, communication security, and collaborative trust mechanisms (Zhang et al., 2026). In addition to AI-related vulnerabilities, the increasing integration of digital technologies into energy systems requires robust cybersecurity mechanisms that protect both operational data and critical infrastructure components. Combining AI with advanced cybersecurity technologies may offer new opportunities to strengthen smart grid resilience, but it also requires careful system design and governance mechanisms. Overall, the integration of artificial intelligence into smart grid infrastructures creates both significant opportunities and new cybersecurity challenges. Understanding these challenges is essential for designing secure and resilient AI-enabled energy management systems that support the reliable operation of future smart grids.

## 3 Methodology

### 3.1 Research design

This project adopts a Design Science Research (DSR) approach to develop prescriptive knowledge addressing data security challenges in AI-enabled energy management systems within smart grid environments. DSR is widely used in information systems research to create artifacts, such as conceptual models, frameworks, and design principles, that address complex socio-technical problems (Hevner et al., 2004; Peffers et al., 2007). Rather than focusing solely on explanation or prediction, DSR emphasizes developing solution-oriented knowledge to guide the design and implementation of information systems. Hence, following established DSR methodology, this paper focuses on the early stages of the design science process, particularly the problem identification and design knowledge generation phases. These phases involve identifying relevant challenges within a problem domain and synthesizing existing knowledge to derive conceptual artifacts that can inform system design (Gregor and Hevner, 2013). In the context of this project, the artifact developed is a conceptual model that categorizes data security challenges in AI-enabled energy management systems and identifies design principles to address them.

### 3.2 Artifact development

**Figure 1:**
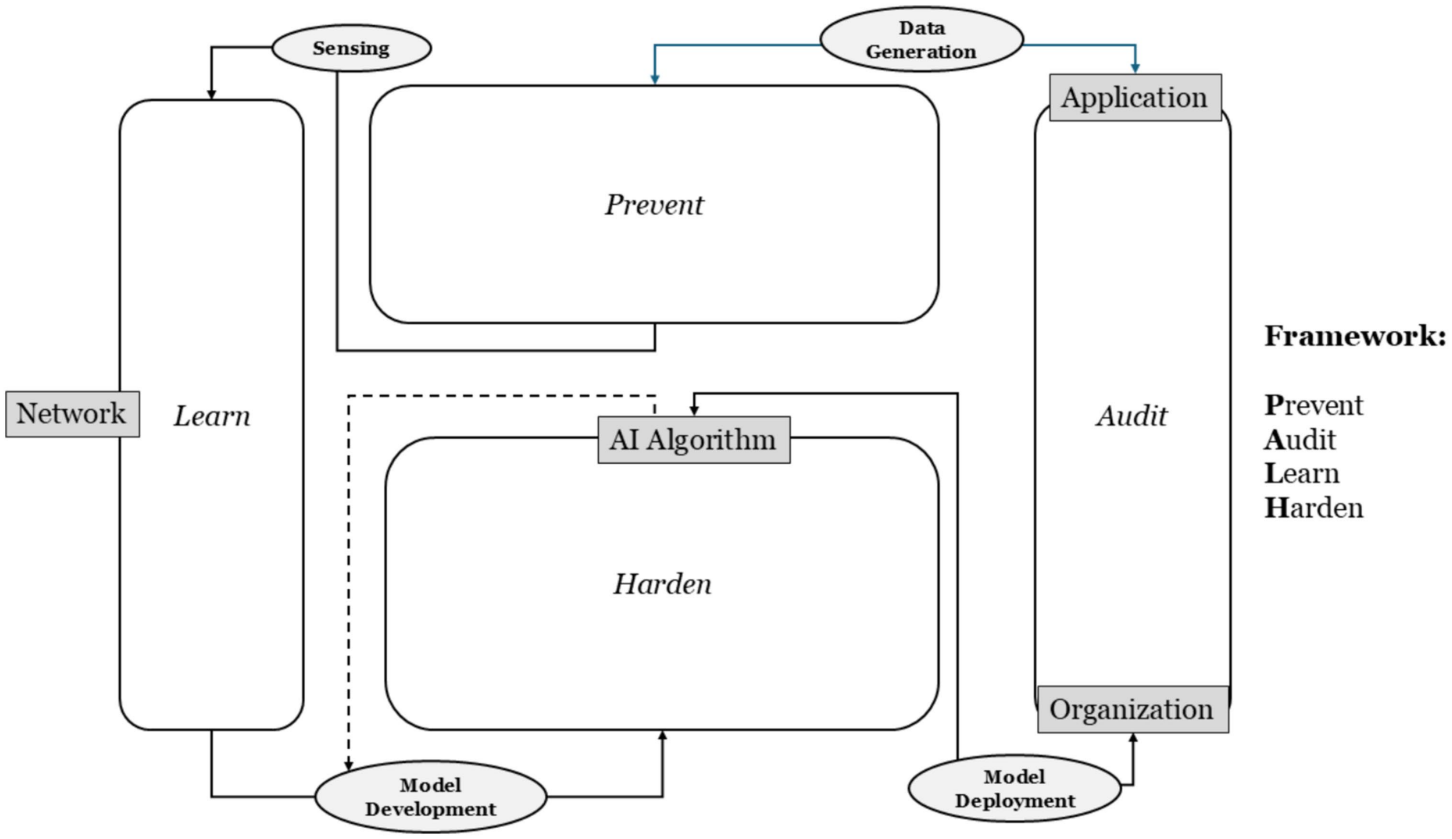
Proposed design framework.

The framework shown in Figure 1 and discussed in detail in what follows is the artifact we developed as part of the design science research (DSR) process to instantiate and communicate the conceptualization of the problem space. The authors followed established DSR guidelines, and the artifact was treated as a design representation that embodies both theoretical constructs and design knowledge and core prescriptive knowledge (Gregor and Hevner, 2013).

The development process began with a structured conceptualization of the problem space, which is AI vulnerabilities (e.g., data poisoning, adversarial attacks, and model inversion) that occur in the data/sensing layer of AI-enabled energy management systems, following the approach suggested by Maedche et al. (2019). We then identified key entities, relationships, and boundaries, and iteratively translated them into a visual form. This translation was done to produce a coherent representation that captures the problem’s complexity while remaining interpretable and actionable. The resulting artifact presented in Figure 1 functions as a boundary object, bridging abstract theoretical understanding and concrete design instantiation.

Consistent with the problematization perspective of (Chatterjee and Davison, 2021), the artifact was not designed merely to address an identified gap, but to critically reflect on the examined problem framing. This allows for a clear articulation of the artifact’s objective, a clear understanding of the stakeholders involved, and an understanding of data security issues in AI-enabled energy management systems. From this, an artifact was constructed to provide clear prescriptive knowledge in the form of a design framework and steps to follow to address identified problems. More so, to realize the artifact, design decisions, such as the structuring of elements, visual hierarchy, and symbolic representation, were guided by the objective of providing a compelling and potentially novel way of understanding the problem domain and our prescriptive knowledge. Moreover, the artifact was developed iteratively, incorporating design science research cycles of refinement to improve clarity, coherence, and alignment with the underlying conceptual model. Throughout this process, considerations were made to ensure that the visual representation effectively communicates relevant relationships and supports reasoning about the problem and the solution.

Finally, following Tuunanen et al. (2024) the artifact development was informed by multiple design echelons. At the instantiation level, the theoretical background discussed in Section 2 represents a concrete visualization of the problem space. Also, the design science research and the core prescriptive knowledge on the right side represent a more abstract level and encapsulate transferable design knowledge on how complex conceptual structures can be visually represented. This dual role supports both practical applicability and theoretical contribution within the DSR framework.

### 3.3 Identify the problem and motivate

In this section, we discuss the design principles identified through the analysis of the academic and practice-oriented literature for specific challenges that require actions at different system layers by specific actors and the need for a set of dedicated tools (see Table 1). We identify problems with current energy management systems and the requirements for transitioning to AI-enabled EMS, which together form the basis of the proposed design principles.

**Table 1:** Design principles their challenges, actions, layers of intervention, actors, and tools.

Design principles (DP)	Challenges	Actions	Layers of intervention	Actors involved	Tools
DP1—Provenance aware data ingestion	Data poisoning	Reduce and prevent	Data/sensing	Data engineers	Public key infrastructure (PKI)
DP2—Privacy-preserving data decentralization	Model inversion	Federate learning	Network/storage	Cloud engineers	Secured multi-party communication
DP3—Adversarial robustness testing	Cyber attacks	System hardening	Model/algorithms	Data scientists AI security auditors	Pyomo
DP4—Explainable security (X-security)	AI decisions	Auditing	Application/human	Control room managers	Human-in-the-loop dashboard

The power grid is currently undergoing an innovative reconstruction to achieve a more decentralized grid infrastructure. This is sometimes referred to as the Smart Grid, which requires current EMS to evolve from model-free methods, such as rule-based systems that rely on dynamic programming (Kindong et al., 2025), to model-based, adaptive, data-driven platforms that integrate real-time monitoring, predictive analytics, distributed optimization, and autonomous decision-making in AI-enabled EMS. Thus, current EMS must deploy advanced sensing infrastructure and adopt interoperability protocols that enable heterogeneous data flows and exchanges, to allow smart grid stakeholders, both old and new, to interact with the grid. As electricity is an essential resource – nobody would want to be without it – it is important to secure the grid. Thus, AI-enabled EMS must first address current limitations, including insufficient interoperability, data quality issues, cybersecurity vulnerabilities, and the black-box nature of AI algorithms. This is crucial in addressing the overarching tension in the evolution of the grid’s decentralization as it aims to become a smart grid. Since becoming smart requires a strong need to share data and information. In the case of the power grid, this means addressing security vulnerabilities stemming from data poisoning and data qualities that complicate the design, development, and deployment of secure, resilient, and autonomous AI-enabled smart grids. This motivates the need for design principles that address and lead to the first identified design principle (DP) in Table 1: *DP1 – Provenance-Aware Data Ingestion.*

Furthermore, technological innovations have significantly impacted the grid infrastructure, opening it to external actors at both the organizational and individual levels. It is a fact, indeed, that consumers of electricity with, for instance, solar panels, have started producing their own electricity, becoming prosumers, meaning they both produce and consume electricity, leading to data decentralization and consequent new challenges for privacy and cyber-attacks. This leads to the second identified design principle in Table 1: *DP2 - Privacy-Preserving Data Decentralization*.

Nowadays, these prosumers, as a next step, invest in batteries. However, to really use these investments efficiently and effectively, an AI-enabled EMS is needed. Previously, EMS in the smart grid was described as a system for monitoring, controlling, and optimizing electricity production and consumption, relying on a model-free approach. AI-enabled EMS must ensure that the overall smart grid is robust enough to withstand cyberattacks, including adversarial AI/ML attacks. This leads to the third identified design principle in Table 1: *D3 - Adversarial Robustness Testing*.

As previously noted, advances in technology have triggered new actors not only at the individual level but especially at the organizational level, including aggregators and EMS providers. The aggregators build their business on collecting data on production and consumption, which they then provide to Distribution System Operators (DSOs) and Transmission System Operators (TSOs). The EMS provider plays an important role in this by developing and implementing EMS as an interface between the utility and the electricity users (prosumers). As current EMS transition to AI-enabled EMS, they must address the black-box nature of AI models, since smart grids require transparency in decision-making, to make the overall grid infrastructure more open. This creates tension between the benefits of decentralization and the security risks posed by incorrect AI predictions, biased decisions, and privacy breaches associated with smart meter data. Therefore, AI-enabled EMS requires continuous auditing of the smart grid system and validation of AI decisions, regulatory compliance, and data handling. This motivates the fourth identified design principle in Table 1: *DP4 – Explainable Security (X-Security).*

Taking the above principles into account, the objective of this project is to develop a robust, secure, and transparent data-to-AI framework that supports a trustworthy AI-enabled energy management system and addresses the identified problem space (data issues). Hence, the objective of the proposed framework is to address the full data lifecycle, from acquisition to model deployment, while explicitly incorporating mechanisms for continuous learning, accountability, and system resilience. Thus, the framework discussed in the following section is designed to instantiate and implement the design principles presented in Table 1 to reconcile key stakeholder requirements, particularly those between data poisoning, adversarial attack, and model inversion, and privacy and security, by embedding these considerations directly into the system architecture.

### 3.4 The artifact

The proposed design framework shown in Figure 1 adopts a lifecycle perspective, with data flowing through interconnected phases of *data generation*, *sensing*, *model development*, and *deployment*. Each stage is augmented by iterative control mechanisms that ensure system integrity and continuous improvement. Moreover, the proposed framework is grounded in a combination of machine-learning lifecycle assurance, socio-technical systems theory, and secure distributed intelligence. These theoretical foundations were discussed in Section 2, which provided the justificatory knowledge necessary to support design choices embedded within the framework.

At the core of the framework is a cyclical process comprising four interdependent *mechanisms* (Gregor et al., 2020): *Prevent*, *Audit*, *Learn*, and *Harden* (PALH), implementing the suggested design principles shown in Table 1. These mechanisms operate continuously across the data pipeline rather than as discrete, sequential steps. Hence, the first objective is to ensure the integrity, quality, and security of data at the point of origin and during transmission across the network. Data is generated and sensed through engineered systems, where data engineers play a critical role in validating, preprocessing, and managing data flows. Secure communication protocols and access controls are implemented to protect data in transit across networks. This stage establishes a trusted data foundation, minimizing the risks of data corruption, unauthorized access, and privacy breaches. The second objective is to enable continuous learning as data moves through the system. Data stored in centralized or distributed repositories is used in an iterative way to train and update machine learning models. Feedback loops from operational environments are incorporated to refine model performance over time. This adaptive capability ensures that the system remains responsive to changing conditions, improving both predictive accuracy and operational efficiency. The final objective is to enhance the robustness and security of machine learning models. The framework includes safeguards against emerging threats such as model inversion and data extraction attacks. Privacy-preserving techniques, including differential privacy and secure training approaches, are considered means of protecting sensitive information. Models are subjected to rigorous validation, stress testing, and adversarial evaluation to ensure reliability under uncertain, potentially hostile conditions. The third objective is to enhance the robustness and security of models. The framework includes safeguards against emerging threats, such as model inversion and data-extraction attacks. Privacy-preserving techniques, including differential privacy and secure training approaches, are considered means of protecting sensitive information. Models are subjected to rigorous validation, stress testing, and adversarial evaluation to ensure reliability under uncertain, potentially hostile conditions. The second objective focuses on ensuring transparency and accountability throughout the data and model lifecycle. The framework incorporates traceability mechanisms, enabling stakeholders to track how data is processed, stored, and used during model development. Algorithmic auditing is conducted by domain experts to evaluate model behavior, fairness, and compliance with regulatory and ethical standards. Documentation, logging, and explainability tools are integrated to enhance interpretability and support informed decision-making. Furthermore, the proposed framework primarily focuses on lightweight AI models (e.g., machine learning and reinforcement learning) that can achieve sufficient accuracy at significantly lower computational cost than large language models (LLMs) and heavyweight models in certain EMS applications. Therefore, the choice of algorithm should consider factors such as computational complexity, inference latency, scalability, memory requirements, hardware compatibility, and lifecycle energy consumption. Integrating these criteria into EMS design strategies is essential to ensure that AI adoption contributes not only to operational intelligence but also to overall system sustainability.

Moreover, given the interdisciplinary nature of smart grid cybersecurity, the problem identification phase is supported by a scoping literature review that systematically explores existing research across multiple domains, including smart grid technologies, artificial intelligence, cybersecurity, and information systems. In what follows, we apply the framework to an application vignette concerning nuclear energy, a prospective, high-stakes scenario for an AI-enabled energy management system connected to smart grids.

## 4 Vignette for application scenario

The Nordic nuclear energy sector, comprising five power plants across Sweden and Finland and operated by organizations such as Vattenfall, Fortum, OKG, and TVO (Sutfeld and Thore, 2025), plays a critical role in the regional energy supply, contributing a substantial share of electricity generation. Despite its strategic importance, the sector remains constrained by highly regulated operating environments, strict data governance, and limited digital integration. Current practices rely heavily on manual processes, siloed data systems, and relatively simple analytical tools, limiting the ability to fully leverage growing volumes of operational data for proactive decision-making. A key limitation lies in the limited data and infrastructure. Regulations require that all data processing and computational resources remain on-premises, with no reliance on external cloud services, and that access to data is governed by strict classification schemes. These constraints hinder the deployment of advanced AI solutions, particularly those requiring large-scale data integration and computational flexibility.

Thus, on-premises AI solutions have emerged as a viable pathway for digital transformation in Nordic nuclear plants. Initiatives such as the AI SNAP project highlight the potential of integrating AI capabilities, including predictive analytics for maintenance forecasting, machine learning models for operational optimization, and large language model (LLM)-based systems for document search and decision support, within secure, local infrastructures. These solutions aim to enhance operational efficiency and support decision-making in non-safety-critical contexts while complying with regulatory requirements, including the EU AI Act (EU, E., 2024) and the NIS2 Directive (European Commission, E. C., 2022), as well as the scope and tasks of pre-trained AI models defined by the provider, such as Meta’s Lama2 (Sutfeld and Thore, 2025). However, adopting on-premises AI introduces new limitations. These include data fragmentation across security domains, limited interoperability between systems, high computational and infrastructure costs, and challenges related to model transparency, validation, and user trust. Furthermore, strict governance procedures slow down deployment, while organizational readiness and skills gaps constrain effective use. As a result, there remains a gap between the promised capabilities of AI and its practical integration in real-world settings.

To address data security challenges arising from potential on-premises AI, our proposed framework, *Prevent*–*Audit*–*Learn*–*Harden* (PALH), can guide the design and deployment of on-premises AI in nuclear plants. In this context, prevention focuses on safeguarding data integrity and access in highly regulated environments; auditing ensures that AI outputs comply with safety, transparency, and accountability requirements before use in accordance with established regulations and license agreements; learning captures both model adaptation and organizational sensemaking as stakeholders interact with AI systems; and hardening strengthens models and infrastructure against risks such as data leakage and adversarial threats. Thus, the real-world implementation of AI-enabled EMS of the Nordic nuclear power plant can adopt our proposed framework, as shown in Table 2.

**Table 2:** Flows, AI process, framework mechanisms, purpose, and key controls and actions.

Flow	AI process	Framework mechanism	Purpose	Key controls/actions
Data generation	Data from sensors, logs, and inspections	Prevent (data poisoning)	Ensure data integrity and trustworthiness	Access controls, data validation, secure on-premise storage, controlled pipeline
Sensing	Real-time monitoring (e.g., vibration, computer vision)	Learn	Improve system understanding and performance	Continuous data feedback, model updates, pattern refinement
Model development	Training predictive models and LLMs	Harden	Protect models from attacks and leakage	Restricted access, secure training environments, privacy-preserving techniques
Model deployment	Decision-support systems for operators	Audit	Ensure reliable and compliant use of AI outputs	Model evaluation, transparency checks, human validation, regulatory compliance

The *data generation* phase, linked to the *prevent* step of the framework, ensures that operational data is continuously collected from sensors, maintenance logs, and visual inspections. Given the critical nature of the nuclear plant, this flow is governed by strict access controls and validation protocols to *prevent* data poisoning. Also, data integrity checks, secure on-premises storage, and controlled data pipelines ensure that only verified, trustworthy data enters the system.

This is followed by *sensing*, which enables real-time sensing (e.g., vibration monitoring and computer vision). This flow allows the system to detect anomalies and patterns in grid operations. These inputs feed into iterative *learning* processes, where models are updated based on new observations and feedback from past decisions. Learning thus occurs both technically (model updates) and operationally (refinement of thresholds and patterns).

The next phase is *model development*, which involves developing predictive models and LLM-based components and refining them using historical and real-time data. During this phase, model *hardening* mechanisms are applied to protect against risks such as model inversion and information leakage. Techniques such as restricted access, controlled training environments, and privacy-preserving methods ensure that sensitive system knowledge cannot be extracted from the models.

Finally, the *deployment* phase enables the integration of AI outputs into decision-support systems used by operators. Before action is taken, outputs undergo *auditing*, during which models are evaluated for reliability, transparency, and compliance with regulatory standards. Human operators validate recommendations to ensure alignment with operational protocols and mitigate the risk of over-reliance on AI.

In summary, the vignette scenario shows how adopting the framework provides a structured approach to bridging the gap between experimental AI capabilities and the socio-technical factors of deployment in Nordic nuclear power plants, and offers a practical pathway to on-premises AI deployment.

### 4.1 Vignette for application scenario

The above vignette provides a narrative illustration of the potential of the PALH framework. In order to further support its theoretical validation (Polos et al., 2009; Teubner et al., 2023; Za et al., 2018), we have run a simulation of the application of PALH to the vignette scenario on two different AI platforms, Claude Sonnet 4.6 and ChatGPT-5.5, whose results are summarized in the following Sections.

#### 4.1.1 Simulation of the vignette scenario with Claude Sonnet 4.6

The simulation considered the actions of two engineers: one who used the PALH framework (Engineer B) and the other who did not (Engineer A). The overall simulation has been structured around five incident phases in which both engineers face an identical, evolving threat and attack scenario, with the same intensity and threat count. Furthermore, each phase captures a decision point as detection, response, containment, and recovery. The shared attack scenario was applied to both engineers simultaneously, and their responses eventually diverged naturally. Finally, all phases have been run at a high attack intensity (70–100%) with 5–6 threats in order to produce a gap between the two engineers’ action outcomes.

**Figure 2:**
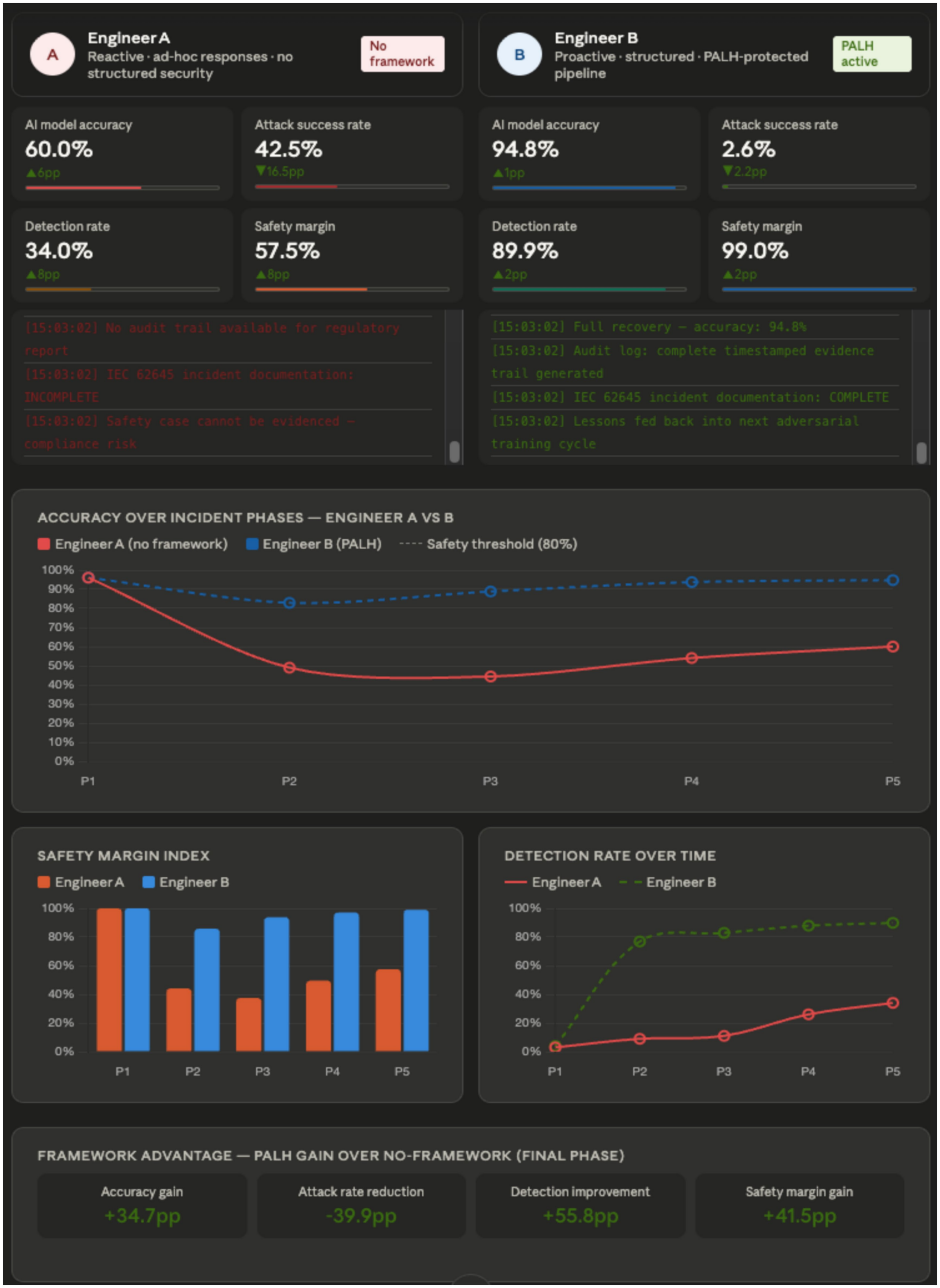
Comparison of the simulation scenarios in Claude Sonnet 4.6 (generated by the system).

In terms of results, Phase 1 is where the most relevant difference occurs, for Engineer A detects the attack only after operators manually flag abnormal readings, with a significant delay and no automated alert. Whereas Engineer B used the Audit layer of PALH within seconds, and the Prevent PALH layer allows for substituting safe values without any human intervention. The AI stays online for Engineer B throughout. Figure 2 shows the contribution of PALH in terms of *accuracy* gain (+34.7 percentage points, pp), *attack rate reduction gain* (−39.9 pp), *detection improvement* gain (+55.8 pp), and *safety margin difference* gain (+41.5 pp).

In particular, it is worth noting that the simulation showed a recovery time and an audit trail completeness differential, where Engineer A did not produce the incident documentation required by nuclear safety standards (e.g., International Electrotechnical Commission - IEC 62645 compliance, safety case documentation, designed to be highly compatible with international nuclear guidelines, such as those published by the IAEA-International Atomic Energy Agency, including Nuclear Security Series-NSS17-T and NSS42-G) while Engineer B actually can and did.

#### 4.1.2 Simulation of the vignette scenario with ChatGPT-5.5

The simulation compared two scenarios for two engineers. As in the previously discussed simulation, one used the PALH framework (Engineer B, Scenario B) and the other did not (Engineer A, Scenario A). Besides the difference in use of the PALH framework, both engineers operate in the same nuclear plant, use same AI monitoring model, face the same cyberattacks, under the same operational conditions. Accordingly, the first Scenario (A) represents conventional on-premises AI deployment where Engineer A trusts sensor inputs, no anomaly filtering, no adversarial hardening, and no audit monitoring, and the AI model is trained only on normal data. On the contrary, the second Scenario (B) for Engineer B represents a PAHL-enacted secure deployment where the “Prevent” layer allows for validating inputs, the “Learn” layer allows for adapting to hostile conditions, the “Harden” layer allows for improving robustness, and the “Audit” layer allows for continuously monitoring of anomalies. The comparison of the two scenarios is shown in Table 3 and the main insights are discussed in what follows.

**Table 3:** Comparison of the simulation scenarios in ChatGPT 5.5.

Metrics	Without PALH	With PALH
RMSE under attack	High (11.4)	Lower (4.0)
Attack success rate	High	Reduced
Detection capability	None	High
Operational stability	Poor	Improved
False alarm awareness	None	Logged
System resilience	Weak	Strong

In phases of normal operations for the two scenarios, both engineers train AI on clean reactor data and achieve similar baseline performance in terms of prediction accuracy and Root Mean Square Error (RMSE = 2.1). The difference emerged when cyberattack begins with false reactor temperature readings, manipulated neutron flux, reduced cooling flow values. In Scenario A, with the absence of PALH, Engineer A receives misleading operational insights, with AI trusting malicious data, and predictions becoming unstable. Whereas, in the Scenario B, using PALH allowed Engineer B to detect abnormal values (through the “Prevent” layer), flagging suspicious activities (through the “Audit” layer), while hardened AI resisted perturbation.

## 5 Conclusion

This article presents the first steps in a Design Science Research (DSR) project aimed at developing actionable design knowledge to secure AI-enabled Energy Management Systems. The transformation from a traditional grid to a smart, digitalized grid highlights the integration of advanced technologies, such as artificial intelligence (AI). Integrating AI into EMS transforms smart grids into highly complex cyber-physical and socio-technical systems (Ibrahimli et al., 2026). It can be concluded that this introduces new data security and governance challenges. This in turn motivates the DSR project in how to develop an AI-enabled cyber-physical system, such as an AI-enabled EMS, that has a significant potential for enhancing resilience in smart environments. The starting point of the DSR project was the following research questions: *What security issues are of concern when developing and implementing AI-enabled energy management systems?* And: *How can they be addressed through prescriptive design knowledge?* Then, in the article, we present an artifact addressing it in the form of a proposed design framework. This latter adopts a lifecycle perspective, with data flowing through interconnected phases of *data generation*, *sensing*, *model development*, and *deployment.* The developed artifact is a conceptual model that categorizes data security challenges in AI-enabled EMS. The framework is based on four design principles identified through the analysis of academic and practice-oriented literature. These design principles are developed to address specific challenges. A concluding remark is that these specific challenges require actions at different system layers by specific actors and the need for a set of dedicated tools The main conclusion is that the developed framework *Prevent*–*Audit*–*Learn*–*Harden* (PALH), can guide the design and deployment of AI-enabled EMS, and by that deal with security issues that can be a fact when implementing AI-enabled energy management systems in the transition to a decentralized smart grid. The framework design principles provide an initial, integrated framework for managing security in AI-enabled EMS, also considering high-stakes environments like the one described in the vignette. Nevertheless, the current proposal presents limitations in the fact that the artifact presented is the paper is the results of the first iterations of DSR from problem analysis to design and development (Tuunanen et al., 2024), with a demonstration limited to suggestions from a vignette scenario, thus supporting the artifact mainly at the conceptual level. Future work includes additional DSR iterations to elaborate on the identified design principles, the development of testable propositions, and their instantiation in a prototype, evaluated and demonstrated through case studies and primary data from field studies, focus groups, and interviews. Notwithstanding these limitations, we argue that the proposed artifact, besides a theoretical contribution intertwining different streams of research in AI, energy management, and information systems security, also provides strategic points of attention and orientation for practitioners aiming at having a systemic and integrated approach to secure the integration of AI in energy systems as well as a view on key axes for the definition of policies acting at multiple layers of the energy ecosystems and infrastructures.

## Data Availability

The original contributions presented in the study are included in the article/supplementary material, further inquiries can be directed to the corresponding author.
